# Are Radiation Target Volumes for Postmastectomy Radiation Therapy Too Large? Initial Report of the Complication Avoidance of Reconstruction Implant Radiation Therapy (CARIT) Study

**DOI:** 10.3390/curroncol30020175

**Published:** 2023-02-14

**Authors:** Aruni Jayatilaka, Ashira Lokhandwala, Kimya Manouchehri, Muriel Brackstone, Michael Lock

**Affiliations:** 1 Schulich School of Medicine and Dentistry, Western University, London, ON N6A 3K7, Canada; 2 Department of Oncology, London Regional Cancer Program, London, ON N6A 5W9, Canada; 3 Division of General Surgery, London Health Sciences Centre, London, ON N6A 5W9, Canada

**Keywords:** postmastectomy radiation therapy, clinical target volumes, capsular contracture, cosmesis

## Abstract

Following mastectomy for breast cancer, women may choose implant-based reconstruction for many reasons, such as cosmesis, self-identity, and the ability to wear particular items of clothing. However, postmastectomy radiation therapy (PMRT) can compromise these cosmetic goals, including as much as a 40% loss of implant rate. To minimize the risk of radiation toxicity, it is important to consider how clinical target volumes (CTVs) can be optimized in PMRT to preserve the implant and reduce complications. Typically, guidelines from organizations such as the Radiation Oncology Group are used, which include regions previously encompassed by tangential fields. This includes all structures below the pectoralis muscle, such as the chest wall, where the risk of recurrence is negligible; this technique often requires incidental inclusion of portions of the lung and heart plus circumferential radiation of the implant. We present the preliminary single institution case series of a technique of complication avoidance of reconstruction implant radiation therapy, called CARIT, where the chest wall, and a large proportion of the implant, is not irradiated. In a retrospective review of 30 cases in which CARIT has been attempted, it was found that 24% of patients treated required a second surgery due to Baker grade III/IV capsular contracture. Using the Modified Harvard Harris Cosmetic Scale, 66.5% of patients had cosmetic outcomes rated as “good” or “excellent”. CARIT could offer a technique to reduce complications in postmastectomy implant-based reconstruction patients, with our next steps focusing on improving dosimetry, and formally comparing the cosmesis and tumor control aspects with commonly used techniques.

## 1. Introduction

Breast cancer management requires a combination of modalities that have improved survival, but may result in cosmetic complications and the need for additional surgery [1,2,3,4,5]. Following the removal of breast tissue, women may opt for autologous (involving tissue from abdomen or back) or implant-based (silicone or saline inserts) reconstruction [6]. The adjuvant radiation therapy field typically includes entire chest wall, which can lead to radiation related toxicities for the lungs and heart, such as pleural effusion, pulmonary fibrosis, and pericardial disease [7,8]. When combined with immediate implant-based reconstruction, PMRT has been associated with complications such as infections, implant exposures, hematomas, and most commonly, capsular contracture [9], with a high incidence of major corrective surgery being required for implant-based reconstruction compared to autologous tissue-based reconstruction [10,11]. Therefore, it is important to consider how clinical target volumes (CTVs) can be optimized in PMRT to avoid intercostal muscles, ribs, and lung in order to preserve the implant and reduce complications.

For mapping CTVs of the breast, chest wall, and regional nodes, there are two international consensus guidelines that are commonly used: the Radiation Therapy Oncology Group (RTOG) and the European Society of Radiotherapy and Oncology (ESTRO) [12]. The RTOG atlas includes the breast and chest wall for PMRT, with the chest wall encompassing the pectoralis muscles, intercostal muscles, and ribs [13]. In contrast the updated ESTRO atlas only includes the skin and subcutaneous levels of the chest wall and excludes the pectoralis major muscle, intercostal muscles, and ribs [14]. For the delivery of PMRT following an implant-based breast reconstruction, the ESTRO guidelines recommend the following three CTVs: the space between the skin and superficial sides of pectoralis muscles, the region of the implant that is not covered by muscle on the ventral aspect, and the space between the dorsal surface of the implant and pectoralis muscles [14]. This volume-based radiation therapy protocol minimizes any unnecessary radiation to the implant, lungs, and heart while still providing radiation to tissues at risk of recurrence. It was reported by Lao et al. (2021) that local recurrences following PMRT could be stratified into those in the skin/subcutaneous tissue, pectoralis muscle, or deep chest wall (intercostal muscles, ribs, and subcostal of pleural space) [15]. Their study showed that out of the 26 patients that recurred (from a total of 1571 patients who underwent mastectomy), 20 (78%) had recurrences confined to the skin/subcutaneous or pectoralis muscle levels, and 5 patients (19%) had axillary recurrence alone. These locoregional recurrences represented 1.7% of all mastectomy patients within the database; one patient (3.8% of all locoregional recurrences and 0.06% of all mastectomy patients) presented with a deep chest wall recurrence. A full systematic review was completed within this study, and the data were found to be consistent with other publications addressing the specific anatomical location of failure [15]. Given that few recurrences occurred below the pectoralis muscle, applying the more aggressive RTOG CTV recommendations may be considered overtreatment. Given that technology now allows us to accurately localize anatomy and sculpt the dose away from low-risk structures previously included in standard tangential fields, this is an area that requires further research so that we can determine the ideal balance between the goals of preventing local recurrence and the avoidance of radiation therapy complications.

## 2. Methods

At our institution, a specialized technique called complication avoidance of reconstruction implant radiation therapy (CARIT) was developed based on the ESTRO guidelines. A CTV-Chest was contoured to encompass the tissue ventral to the major pectoral muscle. In regions where the muscle is not present, the ribs and chest wall served as the posterior border. Coordination with the surgeon and use of the operative report and pathology report were recommended. Furthermore, the first 3–5 mm of skin was not considered part of the CTV-Chest. Field borders were the standard recommendations of the RTOG Breast Atlas. As a precaution to ensure standardization and avoid compromising local control, additional anatomical guidance was used. Medial border was lateral to the medial perforating mammary vessels. Lateral border was ventral to the lateral thoracic artery, which is usually ventral to the mid-axillary line. The technique avoids irradiation of the deep chest wall by using a smaller CTV, thereby avoiding the problematic circumferential irradiation of breast implants in PMRT. 

We have completed a retrospective review of the first 30 cases in which CARIT has been implemented. The goal of this retrospective chart review was to better understand the feasibility and identify any unexpected toxicities and/or complications; in particular, any impact on capsular contracture and cosmetic complications. Capsular contracture was rated using the Baker Classification Scale. Cosmesis was rated using the Modified Harvard Harris Cosmetic Scale. According to the scale, “good” is defined as having mild asymmetry or slight difference in the size or shape of the breast when compared with the baseline image; mild reddening or darkening of the breast; and/or the thickening or scar tissue within the breast causing only a mild change in shape. An “excellent” cosmesis is defined as having minimal or no difference in size or shape or consistency of the breast; there may be mild thickening or scar tissue within the breast but not enough to change the appearance of the breast [16]. The Modified Harvard Harris Cosmetic Scale was chosen due to its ease of operation, and for comparing against the ipsilateral postsurgical breast prior to radiation therapy. Based on previous completion rates at our center, “feasibility” was defined as the completion of all aspects of treatment for greater than 90% of patients.

## 3. Preliminary Results

Following initial analysis, it was found that 24% of patients treated with the implant-sparing technique required a second surgery due to having grade III/IV capsular contracture. Cosmetic outcomes were rated as “good” in 54% of all patients and “excellent” in 12.5% of all patients. The average time elapsed between surgery and radiation therapy was 124.9 ± 121 days. Lastly, CARIT is considered feasible, as 24 patients (96%) completed treatment planning and treatment successfully.

## 4. Discussion

Our study’s finding for capsular contracture may be a promising result in comparison to the outcomes noted in literature of the current standard of care treatments. The baseline risk for the primary complications, such as a poor cosmetic or painful outcome, has a wide range in the literature, likely due to variable follow up, inconsistent classification of toxicity methods, and small sample sizes. For example, a well-performed investigation of a large number of patients by Hammond et al. (2021) looking at 451 patients undergoing mastectomy with implant reconstruction found that the rate of capsular contracture was 18.7% in patients receiving PMRT (following standard radiation therapy techniques) [17]. A study by Dicuonzo et al. (2020) found that in a group of 75 patients with permanent implants who received PMRT, 40 patients (53.3%) had reconstruction failure [18], demonstrating the variation in the reconstruction failures to capsular contraction. However, most of the data are consistent in indicating that current PMRT has a significant impact on complications. A systematic review and meta-analysis by Awadeen et al. (2022) found that irradiated breasts were more likely to develop capsular contracture (risk ratio 5.17, 95% CI 1.93–13.80, *p* = 0.001) and lose implants (risk ratio 2.89, 95% CI 1.30–6.39, *p* = 0.009) when reviewing six articles encapsulating 391 breasts that had PMRT [19]. The use of the standard post-op classification systems, such as the Baker system, and trials with larger combined samples may provide the best estimate of complications. A systematic review by Momoh et al. (2014) that pooled data from 26 trials estimated the risk of severe capsular contraction to be 32% (95% CI 20–46%, *p* < 0.0001) [20]. Recent data from the Massachusetts General Hospital in 2020 revealed that the 5-year cumulative incidence of any reconstruction complication was 19.5% for implant reconstructions without PMRT [21]. The addition of PMRT resulted in a 5-year cumulative incidence of 36.8% [21], similar to the pooled point estimate of the baseline risk by Momoh et al., and much higher compared to our reported Baker III/IV scores.

Interestingly, a study by Ho et al. (2012) had explored long-term outcomes in breast cancer patients undergoing PMRT after exchanging tissue expander for a permanent implant, specifically determining the rates of permanent implant removal or replacement (PIRR). They found a 2-year and 7-year rate of 8.0% and 17.1%, respectively, for implant replacement, and 9.0% and 13.3%, respectively, for implant removal [22]. When considering the reasons for the PIRR events, the majority (47%) were attributable to multifactorial etiologies, which included patient or physician dissatisfaction, suboptimal cosmesis, grade III or IV capsular contracture, or a combination of the above [22]. This highlights the importance of factors such as capsular contracture and cosmesis, and how they contribute to the long-term outcomes of the reconstructed breast with PMRT, such as implant failure.

Literature focusing on the cosmetic outcomes, specifically using the Modified Harvard Harris Cosmetic Scale, was limited. Baschnagel et al. (2012) followed up 60 patients (excluding the 19 patients with reconstruction failure) and found that 75% of the cohort were rated as having excellent/good cosmesis and 25% were rated as having fair/poor cosmesis using the Harvard Scale as defined by clinicians [23]. Our results of 66.5% of patients having cosmetic outcomes rated as good or excellent, albeit in a smaller sample size, remains comparable to the findings of Baschnagel et al. A study by Anderson et al. (2009) followed the complications and cosmetic results amongst women who had PMRT to either a temporary tissue expander or permanent breast implant. Cosmesis was scored either as “excellent/good” or “fair/poor” using the Harvard Scale and definitions as defined by clinicians. They found “excellent/good” scores in 80% of the permanent implant group, in a sample size consisting of 12 patients [24]. These studies highlight the current, broad, and limited landscape of cosmesis outcomes following PMRT; our hope is that CARIT can add to the literature regarding cosmesis outcomes—especially in the context of utilizing a method in which one avoids irradiation of the deep chest wall by using a smaller CTV.

Lastly, it is also important to reflect on skin toxicity, an aspect that we will be following through a cohort analysis at acute (<3 months from PMRT) and chronic (1 year from PMRT) timepoints using the National Cancer Institute’s Common Terminology Criteria for Adverse Events (CTCAE) v.4.02. The current literature focuses on factors such as the role of fractionation and dosimetric parameters in skin toxicity following radiation. Parekh et al. (2018) found that the rate of CTCAE grade ≥2 dermatitis and moist desquamation was high in 50 patients treated with PMRT at 48% and 24%, respectively [25]. A study by Pignol et al. (2014) had prospectively evaluated 257 women during and post PMRT. The endpoints included skin toxicity (measured using CTCAE), moist desquamation (where “extensive” was described as desquamation extending well outside skin folds), and pain (measured using visual analog scale). It was found that 84 (32.7%) patients had CTCAE grade ≥3 skin toxicity, 73 (28.4%) patients experienced extensive moist desquamation, and 57 (22.2%) patients had severe pain (defined as pain impacting activities of daily living) [26]. These studies give us an understanding that skin toxicity, specifically moist desquamation, is a factor that is common following radiation therapy. As such, it is important to assess the skin toxicity and comment on moist desquamation, if possible, in the analysis of CARIT going forward.

## 5. Conclusions

This new way of providing PMRT in the treatment of breast cancer through CARIT provides irradiation to the target tissue but spares deep organs to prevent adverse effects. Moreover, for women with implant-based reconstruction, CARIT offers a way to avoid important complications, such as capsular contracture and implant failure, requiring corrective surgery. Potentially, the reduced CTV would reduce the risk of pneumonitis, pain, rib fractures, second malignancy, and cardiac events. It is important to consider both the physiological impacts of PMRT on the implant but also the cosmetic outcomes; as more women opt to undergo breast reconstruction post mastectomy, the appearance of the reconstructed breast can affect women’s quality of life. From our initial analysis, the findings with regards to capsular contracture appear to be comparable when compared to other literature. With respect to cosmesis, the number of studies exploring this aspect in the PMRT setting was limited; however, the initial findings from CARIT appear to be promising when compared against the studies by Baschnagel et al. and Anderson et al., who used the Harvard Scale. We will follow up with a cohort analysis to compare the patients who receive the current standard radiation therapy treatment to the specialized technique to see if the new method is as effective in improving the risk of toxicity, improving cosmesis, and maintaining tumor control. Ultimately, we hope that the findings from this study will help to develop an improved approach to radiation treatment in the breast cancer patient population following mastectomy and reconstruction.

## Data Availability

Data available on request due to restrictions (e.g., privacy or ethical). The data presented in this study are available on request from the corresponding author. The data are not publicly available due to ongoing research and analysis.
